# The global challenge of reducing mercury contamination from artisanal and small-scale gold mining (ASGM): evaluating solutions using generic theories of change

**DOI:** 10.1007/s10646-024-02741-3

**Published:** 2024-03-02

**Authors:** Allison R. Aldous, Tim Tear, Luis E. Fernandez

**Affiliations:** 1 The Nature Conservancy, Calgary, AB Canada; 2 Biodiversity Research Institute, Portland, ME 04103 USA; 3 Sabin Center for Environment and Sustainability, and Department of Biology, Wake Forest University, Winston-Salem, NC 27106 USA; 4 Centro de Innovación Científica Amazónica, Puerto Maldonado, Madre de Dios 17000 Perú; 5 Department of Global Ecology, Carnegie Institution for Science, Stanford, CA 94305 USA

**Keywords:** Mercury contamination, Artisanal and small-scale gold mining (ASGM), Indigenous people and local communities (IPLC), Theory of Change (ToC), Situation model, Amazon basin

## Abstract

Mercury contamination from artisanal and small-scale gold mining (ASGM) currently accounts for 37% of the global total, often affecting tropical regions where regulations, if they exist, are often poorly enforced. Ingestion by people and other animals damages the nervous, reproductive, and cognitive systems. Despite the efforts of many organizations and governments to curb mercury releases from ASGM, it is increasing globally. There are many possible interventions, all with significant complexity and cost. Therefore, we recommend taking an established systematic approach to articulate the current situation and construct theories of change (ToC) for different possible interventions for any government or organization trying to solve this problem. Here we present a high-level situation analysis and generic ToC to support a more coordinated approach that explicitly builds upon previous experience to identify organization- and situation-appropriate engagement on this issue. We then illustrate the use of these generic models to construct a specific ToC with a policy-focused entry point. This includes interventions through (1) engagement with the global Minamata Convention on Mercury; (2) support for existing national laws and policies connected to ASGM and mercury contamination; and (3) engagement of indigenous people and local communities with governments to meet the governments’ legal obligations. By methodically articulating assumptions about interventions, connections among actions, and desired outcomes, it is possible to create a more effective approach that will encourage more coordination and cooperation among governments and other practitioners to maximize their investments and support broad environmental and socio-political outcomes necessary to address this pernicious problem.

## Introduction

**Fig. 1:**
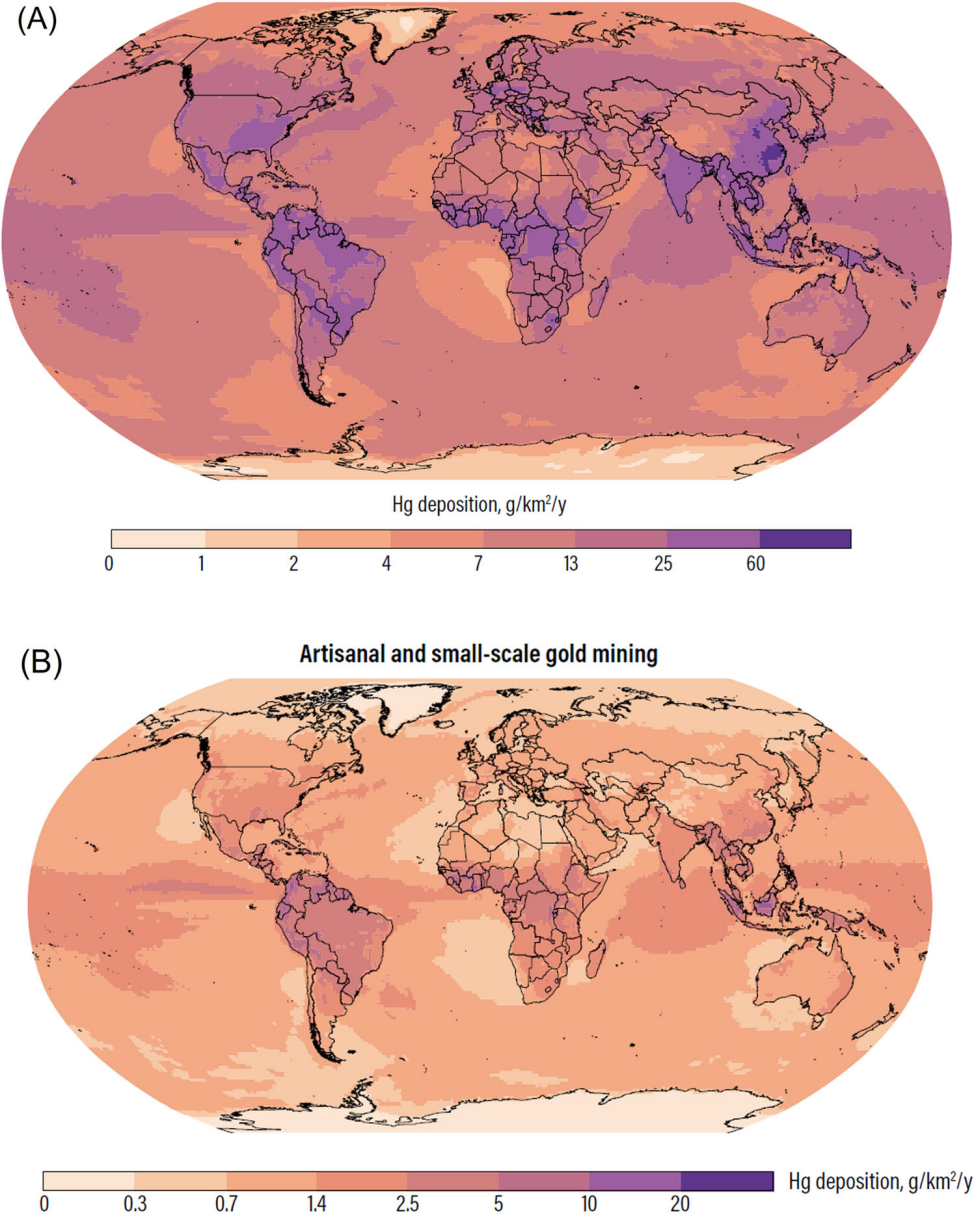
Global deposition of mercury from (**A**) all sources, and (**B**) ASGM. Many areas receive much more significant mercury deposition (e.g., South America, Central Africa, and Southeastern Asia) in comparison to other areas in the world (Adapted from the Global Mercury Assessment, (UNEP UN Environment Programme (2019))). Figures reproduced courtesy of UNEP UN Environment Programme (2019)

Mercury contamination from artisanal and small-scale gold mining (ASGM) has recently become a global conservation and human rights challenge in many parts of the world, negatively impacting both nature and the people closely reliant on intact ecosystems for nutrition, livelihoods, and culture. Less than two decades ago, industrial pollution from power plants was the dominant source of mercury contamination globally. Today, ASGM accounts for 37% of all anthropogenic mercury emissions (UNEP UN Environment Programme (2019)) (Fig. 1).

While estimates vary widely, globally, the artisanal mining sector is estimated to employ 15–25 million people, who support more than 148 million dependents, with much of the mined product being gold (Hilson and Maconachie 2017; Steckling et al. 2017). This sector produces between 380–870 metric tons of gold annually (Cheng et al. 2023), accounting for varying amounts of gold supply. For example, of some of the higher gold-producing countries, ASGM accounts for two thirds of gold from China, one third from Peru, and nearly 100% of gold produced in Colombia (Yoshimura et al. 2021). ASGM also results in deforestation, habitat destruction, soil degradation, increased erosion, and channel dewatering, further stressing fragile ecosystems (Caballero Espejo et al. 2018; Bruno et al. 2020). ASGM often occurs in tropical regions, releasing large quantities of mercury to intact and biodiverse regions such as the Amazon Basin, Indonesia, and the Congo Basin (WWF 2018; UNEP UN Environment Programme 2019). In South America, ASGM accounts for 83% of anthropogenic mercury emissions (UNEP UN Environment Programme 2019).

Mercury is inextricably linked with ASGM. It is used to extract gold from ore and alluvial sediments, typically mixed with crushed or milled ore or with alluvial sediments containing gold to create an amalgam that binds gold particles to mercury increasing gold recovery rates. Because of the inefficiencies and low technical character of ASGM, a high proportion of this mercury is released to the environment, either as vapor or liquid elemental mercury.

Mercury is highly toxic and can lead to serious health problems for miners, their families, and nearby communities (Steckling et al. 2017). A potent neurotoxin, mercury is listed by the World Health Organization as one of its top 10 chemicals of public health concern (WHO 2020). It causes cognitive and neurological impairment and is especially damaging to the developing nervous systems of children and infants (Basu et al. 2018). Because it can pass the placental barrier, women of childbearing age are at particularly high risk of passing the mercury to developing fetuses, resulting in intergenerational impacts to communities.

Mercury contamination has been identified as not only a human health issue but also as a conservation issue. Because of its ability to biomagnify and bioaccumulate, mercury concentrations are typically higher in organisms at higher trophic levels in both aquatic and terrestrial ecosystems (Scheuhammer et al. 2015). Mercury is captured and concentrated in tropical forest ecosystems (Gerson et al., 2022) and in tropical aquatic ecosystems altered by mining (Gerson 2020). Elevated mercury concentrations have been found in several animal taxa in impacted areas, including birds (Pisconte et al. 2023–this Special Issue), bats (Moreno-Brush et al. 2016), fish (Barocas et al. 2023), and insects (Dias dos Santos et al. 2021; Eagles-Smith et al. 2020). Elevated mercury exposure has been found to alter fish and bird reproductive organs, damage tissues, and result in decreased animal size and reproductive output (Evers 2018).

Because of its importance as a food source for millions of people, mercury contamination in fish has garnered particular interest, as fish consumption is the primary pathway for mercury exposure to humans (WHO 2021). Mercury may also be a growing threat to the health of freshwater fish species, which have declined by 84% since 1970, more than any other group of species (Harrison et al. 2018). Mercury contamination can dramatically reduce the reproductive potential of fish (Depew et al. 2012a, b; Evers et al. 2023–this Special Issue). In addition to fish as a source of nutrition, many fish species are culturally important to indigenous people and local communities (Noble et al. 2016).

Although mercury is used in many regulated, legal industrial processes, its use in the largely informal and unregulated ASGM sector means that the global economies supplying mercury to ASGM regions are not well understood (Villar and Schaeffer 2019). In certain countries, organized crime groups have been discovered using gold from ASGM to launder money from other illicit activities, such as drug trafficking, illegal logging, and human and wildlife trafficking (U.S. Federal Bureau of Investigation 2019; U.S. Bureau of International Narcotics and Law Enforcement 2019). The involvement of organized crime in ASGM makes for a mix of human rights abuses, corruption, and violence (Vallejos et al. 2020). Furthermore, vulnerable populations are both impacted by ASGM, but may also be participating in ASGM activities because it can be more lucrative than other livelihoods.

Due to its informal and unregulated nature, ASGM has been a subject of concern for governments, environmental organizations, and human rights advocates. Governments and civil society organizations have worked to develop interventions for mercury threat abatement from ASGM that range widely in scale from local (e.g., mercury-free ASGM technologies; monitoring contamination) to national (e.g., ending the trade of mercury; enforcement of ASGM bans) to global (e.g., mercury-free gold supply chains). As these strategies are generally complicated, expensive, and require long-term commitments, and their impacts are still largely uncertain, a more careful examination of the issue is needed to better align and integrate efforts to address this growing problem more effectively.

### Using Theories of Change to develop interventions for reducing mercury contamination from ASGM

Due to the complex pathways in which mercury enters a country or contaminates any given area, as well as the many factors that influence the effectiveness of ASGM interventions, deciding which interventions are most likely to be impactful presents a multifaceted challenge. Here we argue that a theory of change (ToC), which is a clear and detailed explanation of how and why a particular intervention aims to achieve its intended outcomes and impact, is a powerful approach for assisting in that decision. It describes a causal chain of results, referred to as “Impact Pathways,” which emerge from the activities and outputs of the intervention. These pathways are based on specific underlying assumptions within the context of the situation (Conservation Measures Partnership 2020; Salafsky et al. 2021).

Theories of change have been used for many years across many sectors and are recently gaining much wider use and recognition in the environmental management and conservation sector (Salafsky et al. 2021). Among the benefits are the use of ToC as a tool for facilitating dialogue among stakeholders, identifying the suite of potential entry points for different actors, clarifying the possible strategies and actions needed to implement the strategies, and identifying possible gaps in logic or missing actions for successful outcomes. This work is constructed within a framework for structuring evaluations of effectiveness and impact, a means for connecting intended actions and interventions to desired outcomes, and in some cases for evaluating the return-on-investment of different strategies (Salafsky et al. 2021).

The selection of any intervention operates on the assumption that, when effectively deployed, the intervention will achieve a desired outcome. In this paper, we employ a ToC approach to chart results and impacts across different intervention levels (local, subregional, national, and transnational) (Conservation Measures Partnership 2020). The goal is to determine how these interventions might influence a specific outcome—namely, the reduction of mercury contamination from ASGM.

Salafsky et al. (2021) posit that the development of *generic* ToC that can clearly define strategies and present a template for data and the relationship among actions can be useful to explore the potential effectiveness of conservation strategies and develop an evidence base for its effectiveness. The process of developing generic ToCs should include describing the enabling conditions and the interventions that are to be undertaken, and then along this pathway identify key intermediate results and the ultimate outcomes, assuming the strategy is implemented effectively. The intended value of generating this body of work is to create a “library” that others can benefit from, with the hope that lessons learned from past efforts can help to advance the effectiveness of future activities.

This paper presents a first step towards identifying a generic ToC for working on mercury reduction in ASGM, with a specific focus on “entry points” for governments and civil society organizations. Much of the work on ToCs related to ASGM to date has been part of organizations’ internal program planning, management, and evaluation efforts, for example, individual country ToCs created as part of the Global Environment Facility-funded PlanetGOLD program (GEF 2021). We aim to shed light on some of these earlier approaches and provide an initial framework that can be used by future practitioners to better understand the complexities of mercury in ASGM and accelerate the development of more effective approaches and strategies to mitigate its impacts, and ultimately reduce mercury released into the environment.

The objectives of this paper are to (i) present a high-level situation analysis of mercury contamination from ASGM to identify potential entry points and impact pathways for threat reduction; and (ii) based on this analysis, present ToCs to explore two approaches to risk reduction: policy interventions and integrated empowerment of indigenous people and local communities (IPLC) in policy implementation.

## Methods

**Fig. 2:**
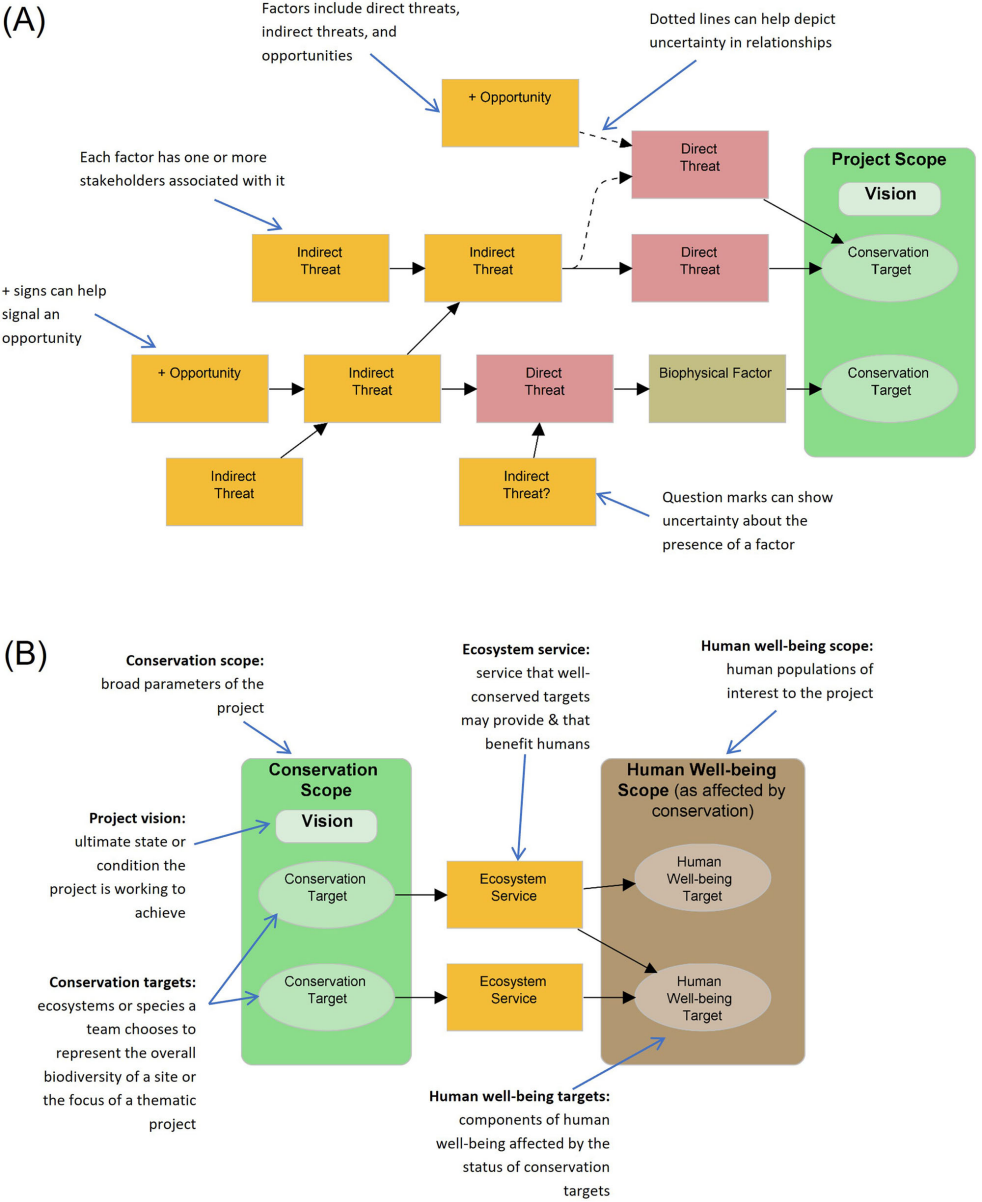
Generic situation models extracted from Conservation Measures Partnership, (2020) showing (**A**) project context and (**B**) scope, vision and targets. Figures reproduced courtesy of CMP (2020)

In this paper, we followed the guidelines of the Conservation Measures Partnership, (2020) and began by developing a situation analysis to illustrate which aspects or factors in the current situation proposed interventions are intending to address. This is done by creating a situation model, which is a graphical or narrative description of the system, including the key stakeholders and influencers, the social, political, and economic drivers, and the biodiversity, human well-being interests, and ecosystem services impacted (Fig. 2). Construction of the situation model is often done from right to left, in other words, by first defining the project scope and vision, then identifying the direct threats and biophysical factors, and finally by clarifying the indirect threats and relationships among them.

From this generic structure of the system, we propose the identification of “entry points” for engagement as this presents an important, scale-dependent distinction that is necessary for identifying appropriate ASGM interventions. We define an “entry point” as a high-level intervention, and a “strategy” as an intervention at a more specific level. For example, entry points can be the introduction of technology or enacting or enforcing policy, with associated strategies being to introduce a specific mercury-free ASGM method and equipment to a particular group of mining communities with the goal of decreasing the use of mercury within a watershed (technology strategy) or banning the import and trade of mercury within a political jurisdiction, with the goal of reducing the local availability of mercury to mining communities (policy strategy).

## Results

**Fig. 3:**
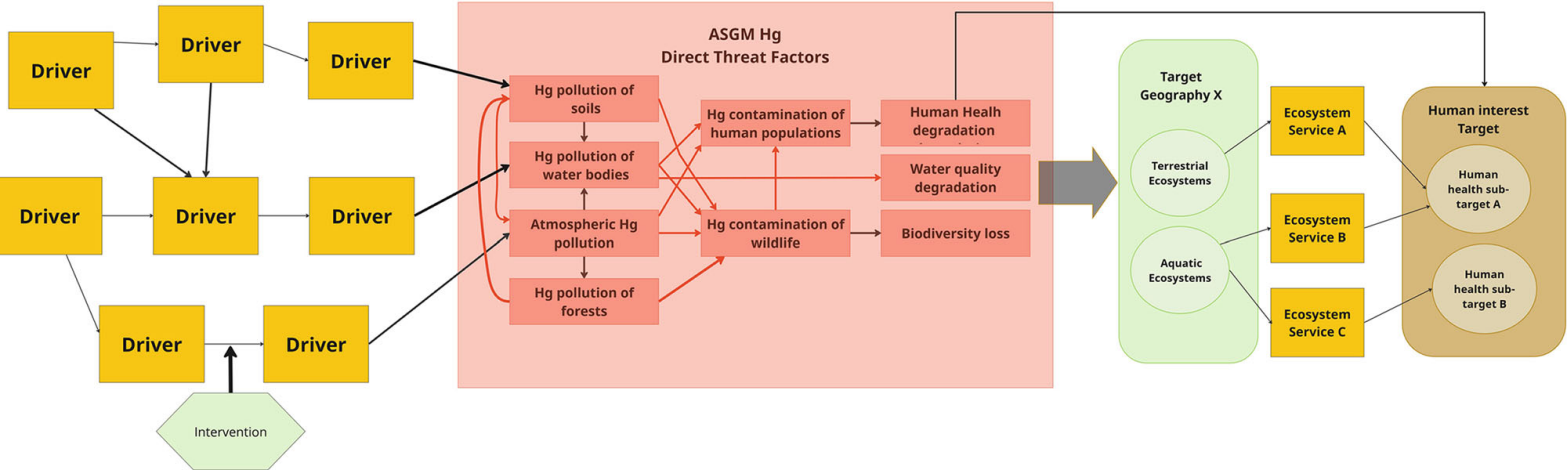
A generic, high-level situation analysis of a policy approach to mercury reduction that includes engagement and empowerment of IPLC in implementation. Boxes labeled “driver” are generic examples and the one generic “intervention” is intended to illustrate how it could break the connection between drivers. Threat factors may contribute to other threat factors, as well as be direct threats themselves. For example, water quality degradation is a stand-alone threat, and it also exacerbates biodiversity loss and human health degradation. Examples of specific interventions that could be used in a more detailed situation models are provided to illustrate how this generic situation analysis could be expanded for use in any given program area. Figure reproduced courtesy of Conservation X Labs

Following the approach outlined by Salafsky et al. (2021) and illustrated by Boshoven et al. (2021), while relying on guidance materials created by the Conservation Measures Partnership (2020), and drawing from the authors’ experiences, we developed a high-level, generic situation analysis for ASGM policy-level interventions (Fig. 3). Here, we incorporated insights from an unpublished impact analysis of Conservation X Labs’ Artisanal Mining Grand Challenge, a four-year initiative that utilized an open innovation model to develop technical solutions and policy shifts in the global artisanal mining sector (CXL, unpublished report) to create this generic situation analysis.

**Fig. 4:**
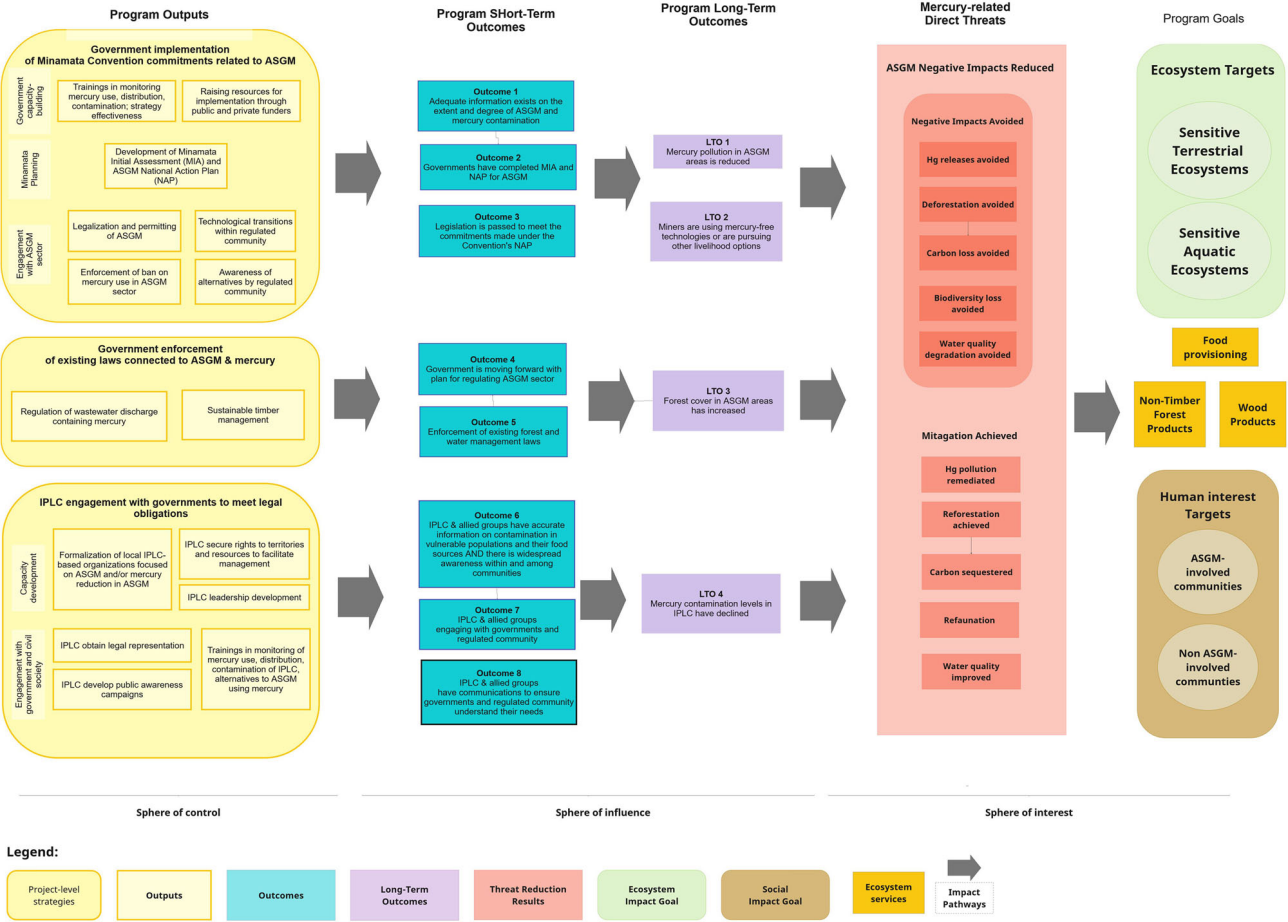
Generic policy ToC to reduce mercury contamination from ASGM activities. Three integrated impact pathways are identified that must be addressed to achieve any program’s goals of improving the health of nature and people impacted by mercury contamination from ASGM activity

From the situation analysis, we formulated a generic ToC for an overarching policy-based intervention (Fig. 4), based on an extensive review of the literature for current approaches being used in countries around the world (BRI and TNC 2022). While policy solutions have not always been adequate (Hilson 2006; Hilson and Maconachie 2020; Lara-Rodríguez and Fritz 2023), government interventions in the sale and trade of both gold and mercury will be required to make any advances in reducing mercury contamination. In our generic ToC, we identify three “entry points” to illustrate this approach, which are presented independent of location or specific time-delineated outcomes. We introduce a critical emphasis – that these three entry points are inextricably linked, and while each has its own “impact pathway”, none of the pathways is likely to be successful without success in the others. This explicit integration of multiple entry points is absent from many proposed policy interventions that focus on a single entry point.

We identified three integrated policy impact pathways: (1) governments more effectively enforce and incentivize compliance of *existing* national laws and regulations on mercury use; (2) governments develop *new* laws and policies according to international agreements such as the Minamata Convention to reduce mercury; (3) and indigenous and other civil society groups hold governments accountable for developing and enforcing laws and policies to reduce mercury releases. Pathways (1) and (2) are intended to all be coordinated under a party’s Minamata National Action Plan.

The three pathways recognize that policy assessment and improvement (including the addition of new policies) are best aligned with the global guidance coming from Minamata Convention obligations. They also emphasize a missing component of many national governmental approaches to policy creation and implementation, namely that local partnerships and engagement in on-the-ground and in-the-water policy implementation is essential to success, as indigenous people and local communities are at the front lines of impact from mercury contamination and are affected by incentive structures that engage them in ASGM.

In this generic ToC diagram, the emphasis shifts from the generic situational analysis (Fig. 3) by adding in more detail on outputs and impacts. Here, we purposefully make a distinction between short- and long-term outcomes, as this has emerged as critical for program planning as well as for structuring monitoring and evaluation efforts essential in any adaptively managed effort. Similarly, multiple mercury-related threat reduction impacts are specified, that collectively are expected to lead to improvement in the associated ecosystem services.

## Discussion

We recognize that there are many potential entry points to reduce mercury in ASGM activities around the world. While not intended to be comprehensive, some of the more common potential entry points currently being addressed are illustrated in Table 1.

**Table 1:** Potential entry points for addressing mercury contamination of people and nature from ASGM activities

Potential entry point	Example engagement^a^
Government articulation of commitments to the international convention specifically addressing ASGM contributions to mercury contamination^b^	Minamata Convention – Minamata Initial Assessments and ASGM National Action Plans https://mercuryconvention.org/en/about
Government enforcement of existing laws connected to ASGM and mercury^a^	Minamata Convention – National Implementation Plans – https://mercuryconvention.org/en/about
IPLC engagement with governments to meet legal obligations^a^	Sinangoe community in Ecuador: https://coicamazonia.org/lucha-historica-de-la-comunidad-ai-cofan-de-sinangoe-por-sus-territorios-y-derechos-como-pueblos-originarios/
Formalization of ASGM sector and gold supply chain reforms	Funding provided by the Global Environment Facility (GEF) Trust Fund (https://www.thegef.org/what-we-do/topics/mercury). Artisanal Gold Council (https://artisanalgold.org/), Planet Gold (https://www.planetgold.org/)
Technological Innovations	Conservation X Labs’s Artisanal mining grand challenge https://www.artisanalminingchallenge.com/ The U.S. Environmental Protection Agency’s program, Artisanal and Small-Scale Gold Mining Without Mercuryhttps://www.epa.gov/international-cooperation/artisanal-and-small-scale-gold-mining-without-mercury
Protected area management and law enforcement	Frankfurt Zoological Society https://fzs.org/en/news/expert-opinion-illegal-gold-mining/
Outreach, education, capacity building at the community level	World Wildlife Fund http://awsassets.panda.org/downloads/healthy_rivers_healthy_people.pdf
Research and Monitoring	Biodiversity Research Institute’s Center for Mercury Studies https://briwildlife.org/hgcenter/; Centro de Innovación Cientifica Amazónica (CINCIA) https://cincia.wfu.edu/en/

^a^List is illustrative and not meant to be exhaustive. Some organizations engage in multiple entry points

^b^Discussed in this paper

In our generic ToC, the first impact pathway recognizes that individual countries will have a variety of existing laws and policies that address mercury contamination. These could be specific to mercury (bans or regulation on use) or more general to heavy metal contamination (e.g., regulation of wastewater discharge). In addition to banning mercury use and ASGM, governments may have policies that seek to formalize and regulate the ASGM sector, including promoting the use of mercury-free technology (Planet Gold 2023). Policies could also be indirectly related to ASGM, including logging regulations in countries where deforestation is often caused by ASGM activity (Caballero Espejo et al. 2018). Enforcement of these laws and policies will require actions focused on technical skills and support, such as early warning systems to collect and synthesize data on spatial and temporal trends in ASGM activity and related deforestation; monitoring of mercury contamination levels in people and wildlife; training for enforcement officials; and access to mercury-free ASGM technologies.

If effective, these actions should lead to increased institutional and technical capacity within regulating bodies, resulting in improved enforcement of existing laws and policies, and ultimately a reduction in mercury pollution of ecosystems and people. However, it is well-established that many environmental policies are only weakly enforced (Hiriart et al. 2011), and so multiple enabling conditions must be present for this pathway to be effective. In addition to institutional and technical capacity, it is essential to have financial support, political will for enforcement, awareness by the regulated community, credible monitoring systems in place, and reasonable livelihood alternatives for mining communities.

The second policy impact pathway is via the Minamata Convention, which entered into force in 2017. The Convention is a multilateral environmental agreement aimed at reducing and ultimately eliminating global mercury pollution. It is named after Minamata Bay on the west coast of Kyushu Island in Japan, where from 1932–1968, the Chisso Corporation’s chemical factory released wastewater heavily contaminated with the toxic form of mercury (methylmercury) into the Hyakken Harbor, contaminating fish in the bay. The local community was highly dependent on this fishery, and it is estimated that 2,252 people were impacted, resulting in 1,043 deaths (Harada 1995). It was not until 1956 that the disease was linked to mercury contamination. ASGM presents a real risk that many “Minamata Bays” are now happening simultaneously around the world. To reverse this increasing risk, much more organized, aligned, and coordinated efforts are needed, thus necessitating the use of a generic ToC to guide these global efforts.

To date, the Minamata Convention has 146 Parties, including many of the countries with significant ASGM activity, and it requires they develop strategies, take actions, and report on progress related to mercury emissions from ASGM and other industries from which mercury is released (e.g., coal fired power plants, chlor-alkali production, batteries). The Convention also provides technical assistance, information exchange, public awareness, and research and monitoring (Minamata Convention 2023).

Once a Contracting Party has ratified the Convention, it may complete a Minamata Initial Assessment (MIA), which includes inventories of mercury and mercury compounds; sources of emissions and releases; overview of structure, institutions, and legislation available to implement the Convention; identification of populations at risk; current understanding by workers and the public; and a plan for implementation of priority actions for reducing mercury in the environment. When MIAs find that ASGM activities are significant sources of mercury, the countries must produce a National Action Plan (NAP) to address this specific source, following Annex C of the Convention. This includes objectives and reduction targets; specific ASGM-related actions to eliminate; steps for formalization or regulation of the sector; estimates of the quantities of mercury used; strategies for promoting mercury reduction, managing the trade of mercury, involving stakeholders, addressing public health, preventing exposure, and educating the affected communities; and a schedule for implementation.

The ToC for this policy pathway involves supporting Contracting Party nations in completing MIAs, developing NAPs where ASGM is significant, and implementing interventions (articulated in NIPs) to ultimately reduce mercury use and contamination. Many of the actions for this pathway are similar (if not identical) to the first impact pathway, including those to secure necessary technical and financial support. In some cases, the national laws and policies may already exist for NAP implementation, as in pathway (1). In other cases, new laws and policies must be developed to address the findings of the MIA and objectives of the NAP. Like pathway (1), enabling conditions must exist or be developed to ensure implementation and enforcement.

Linking national responses to global conventions is a well-known approach (e.g., Convention on Biodiversity). While explicit linkages to participation of indigenous people and local communities is more of an emerging strategy, a decision was adopted at the 5^th^ Convention of Parties in 2023 to strengthen this element. Thus, the third policy impact pathway defines explicit and purposeful engagement with the many IPLC who are directly impacted by ASGM, either by consuming fish and breathing air that is contaminated with mercury from ASGM, or who themselves participate in ASGM. These affected populations can play a powerful role in engaging with governments to meet their legal obligations made under various laws and policies.

Certain enabling conditions of the IPLC sector facilitate their effective participation (Zhang et al. 2023). Strong tenure security gives communities legal standing over the resource in question (e.g., land tenure where ASGM is being practiced illegally on indigenous territories; mineral rights where there is a question of who can mine; fisheries rights for contaminated food supplies). They also require leadership capacity and access to decision-making platforms to have their voices heard. And finally, communities who may see ASGM as a lucrative opportunity need livelihood alternatives that are culturally appropriate and can ensure a reasonable standard of living (Karres et al. 2022; Zhang et al. 2023). This could include sustainable ASGM that is done without the use of mercury and without extensive habitat destruction (Planet Gold 2023).

In addition to engaging directly with governments in decision-making arenas, indigenous people bring important skills and lessons including knowledge of the rivers, lakes, and forests where ASGM takes place and the fish and other affected species and could be important contributors to monitoring systems that must sample these remote areas. There are powerful examples of indigenous people developing guardian patrol programs to track illegal activities within their territories (e.g., in Ecuador: https://coicamazonia.org/lucha-historica-de-la-comunidad-ai-cofan-de-sinangoe-por-sus-territorios-y-derechos-como-pueblos-originarios/ and Brazil: https://www.weareguardiansfilm.com/). Similarly, monitoring the health of local communities could also be an important indicator of the Minamata Convention’s overall impact. For example, decreasing trends in mercury contamination among indigenous people have been documented recently in the Arctic (Adlard et al. 2021). However, not only are there no comparable trend data in the tropics that we are aware of, the increase in ASGM activity suggests that trends could be very different across the tropics, highlighting the need for investing in more baseline monitoring that is also suggested by the Minamata Convention.

In the absence of basic information on the sources and amounts of mercury contamination in a country, and the identification of appropriate interventions to reduce contamination supported by the national and local governments, it is difficult to envision the informed development of appropriate interventions and associated ToCs that will lead to desired outcomes of reducing or eliminating mercury contamination. Supporting the active engagement of countries with the Minamata Convention is one entry point that can work within an existing policy structure to improve the information and planning needed to better address the impacts of mercury contamination from ASGM.

For example, the most recent Global Mercury Assessment (UNEP UN Environment Programme 2019) estimates that approximately 83% of South America’s mercury emissions are from ASGM activity. Peru and Ecuador became parties to the Minamata Convention in 2017, and Colombia followed in 2019 (Minamata Convention 2023). Their participation in the convention has helped to advance collecting information on the sources of mercury contamination, developing plans for appropriate interventions, and establishing mechanisms to track implementation.

Regarding mercury contamination, Colombia released its first national report in 2017 summarizing the results of the MIA process (UNIDO 2017), which concluded the top two sources of mercury emissions *and* releases into the environment came from the mining sector: (1) *gold extraction with mercury amalgamation* (331,551 kg Hg/year, or 55.7%); and (2) *primary metal production excluding gold amalgamation* (159,105 Kg Hg/year, or 26.7%). These two mining sources accounted for a total of 82.4% of all the mercury emissions and releases into the environment, and approximately 95% of all gold mining in Colombia is ASGM (Yoshimura et al. 2021). Regarding mercury release directly into air and water, one factor emerged as the dominant input for both–*gold extraction with mercury amalgamation* (198,931 kg Hg/year or 86.9% for air and 66, 310 kg Hg/year or 80.6% for water). Mining was also estimated to be a dominant driver of mercury releases to soil (66,310 kg Hg/year or 29.8%).

Following this baseline assessment, a second government-backed study revealed significant mercury contamination in the local communities, fish, water and soils of the middle Caquetá River Basin (MOI 2019). This study was the result of concern expressed by the Puerto Zábalo – Los Monos Resguardo to the National Natural Parks of Colombia to better understand the impact of gold mining activity in the Caquetá area and its effects on the fishery resource and the implications for the health of its inhabitants. This report supports the results of the Global Mercury Assessment (UNEP UN Environment Programme 2019) and Colombia MIA (UNIDO 2017) that ASGM activities are the primary source of mercury contamination.

The work needed to develop the plans necessary to address significant sources of contamination and complex strategies to reduce their impacts on people and nature need the support of local and national governments to establish the enabling conditions needed for success, for which the Minamata Convention provides an enabling structure (Minamata Secretariat 2023). In South America, Ecuador submitted its National Action Plan (NAP) to address ASGM-derived mercury contamination in 2019, while Peru also submitted its National Implementation Plan (NIP) to address all sources of mercury contamination in 2019 – one of only three countries in the world to do so.

In the third policy impact pathway that we present, civil society organizations and governments collaborate with IPLC by supporting their endeavors through financing, supporting citizen science, providing data on amounts and sources of mercury contamination, capacity-building, funding for health care services and training on how to recognize the symptoms of mercury contamination, and information on how to make healthier choices to avoid eating contaminated fish. This work is best done following the lead of the communities, due to the social and economic complexity of ASGM in the landscapes in which they live. In the case where the community is participating in ASGM, civil society organizations and governments can support their transition to a more sustainable ASGM or other livelihood options through financing and capacity building for technology transfer.

Developing a situation analysis for detailing a ToC can refine that strategy in several ways. It can assist in identifying the enabling conditions under which strategies are more likely to be effective (e.g., Boshoven et al. 2020). It can help in the development of a monitoring, evaluation, and learning program and in selecting indicators (e.g., Effective Evaluation (EE) component of Minamata Convention). It can be used to identify assumptions and prioritize research needed to test those assumptions. And it can assist in defining and prioritizing the individual actions needed to be taken.

The complex socio-political context of mercury contamination from ASGM points to a need to develop situation analyses and ToC for improving the effectiveness of interventions. By constructing a generic ToC first, we highlight that our emphasis on key policy-related outcomes and impacts can’t be achieved in isolation. In our example, we illustrate that for the policy actions to be successful, there will need to be engagement and participation of local communities, which is a gap in many national policies. Furthermore, we suggest that multiple strategies from different synergistic entry points are needed to be effective in reducing, and ultimately eliminating this pervasive threat to nature and people.

We recognize that developing a generic ToC is simply a tool to facilitate engagement with stakeholders on reducing the threat of mercury contamination. Once a decision is made to adopt the generic ToC to guide decision-making, important next steps include adapting the generic ToC to the specific context and then conducting a return-on-investment (ROI) or tradeoff analysis to shape which entry point or points will be selected, taking into consideration the local, national, and global contexts as well as the capacities of the implementers. Providing additional guidance on how to shape this next step in decision-making is a current gap in the knowledge and understanding about how best to address mercury contamination in ASGM activities around the world, especially considering the substantial variability in data and knowledge and local contexts that shape ASGM activities in different countries.

There are, of course, limitations to the ToC introduced here. The scope of this analysis was on reducing mercury contamination from ASGM, not the reduction of ASGM itself. In addition to contributing to mercury contamination, ASGM has other negative impacts to the environment (e.g., deforestation, soil degradation, erosion, river sedimentation) and to communities (e.g., human rights and labor abuses). More specific actions to address these threats is needed.

Perhaps the most important aspect of the ToC introduced here is the recognition that engagement of indigenous people and local communities is an essential component to the success of any policy approach. These populations are an integral part of the policy entry point’s impact since these communities experience both the health and socioeconomic impacts of ASGM and mercury contamination. We emphasize the role taken by these communities in engaging with governments to help design and implement more effective laws and policies to address the threat of mercury from ASGM. And yet we also recognize there are many factors that result in high levels of social complexity. For example, in the Amazon, some community members may be small-scale gold miners using mercury, some fishers who may oppose the activity, while others are both miners and fishers (Escobar-Camacho and Rosero-López, pers comm). With consistently high gold prices, easily available sources of mercury, and the low-tech method using mercury for extraction, relying on ASGM as an income stream is a reasonable livelihood decision. This complexity only highlights the need to ensure full engagement of these affected communities.

## Conclusions

Mercury use in ASGM is a multifaceted global problem that presents practitioners with several complex challenges. Despite the substantial efforts that have been channeled into the ASGM sector, there remains a conspicuous absence of explicitly defined strategies in the published literature focusing on mercury risk reduction in the context of ASGM, and associated studies that document with evidence the effective implementation of these interventions that result in the actual reduction in mercury contamination.

In this paper, we present a situation analysis for mercury in ASGM based on the experience of practitioners working in this space, as well as an initial effort at developing a ToC for policy-based interventions. The adoption and use of generic ToC offers a promising avenue for addressing mercury in ASGM. ToCs provide a framework that facilitates system description and definition which can foster a shared understanding of the system and enable stakeholders to converge on a unified definition and terminology, which in turn helps to understand system dynamics. Furthermore, generic ToCs such as these can and should be modified for each local context.

By presenting these generic ToCs, our aim is to contribute to the discussion on how to improve efforts to address an increasing global problem that has proven remarkably resistant to change. We suggest that the use of these tools can help to accelerate the development of effective impact pathways, including in the development of National Action Plans, and offer insights into how best to intervene that explicitly link global to local efforts necessary to ultimately reduce the growing impact of mercury contamination to people and nature around the world.
